# Time to Think “Meta”: A Critical Viewpoint on the Risks and Benefits of Virtual Worlds for Mental Health

**DOI:** 10.2196/43388

**Published:** 2023-02-07

**Authors:** Vincent Paquin, Manuela Ferrari, Harmehr Sekhon, Soham Rej

**Affiliations:** 1 Department of Psychiatry McGill University Montreal, QC Canada; 2 Lady Davis Research Institute Jewish General Hospital Montreal, QC Canada; 3 Douglas Mental Health University Institute Montreal, QC Canada; 4 McLean Hospital Harvard Medical School Boston, MA United States

**Keywords:** metaverse, digital media, virtual reality, mental health, addiction, social functioning, virtual, technology, augmented reality, gaming, social media, cognitive, physical activity, behavior, psychological, development, patient, policy

## Abstract

The metaverse is gaining traction in the general population and has become a priority of the technological industry. Defined as persistent virtual worlds that exist in virtual or augmented reality, the metaverse proposes to afford a range of activities of daily life, from socializing and relaxing to gaming, shopping, and working. Because of its scope, its projected popularity, and its immersivity, the metaverse may pose unique opportunities and risks for mental health. In this viewpoint article, we integrate existing evidence on the mental health impacts of video games, social media, and virtual reality to anticipate how the metaverse could influence mental health. We outline 2 categories of mechanisms related to mental health: experiences or behaviors afforded by the metaverse and experiences or behaviors displaced by it. The metaverse may benefit mental health by affording control (over an avatar and its virtual environment), cognitive activation, physical activity, social connections, and a sense of autonomy and competence. However, repetitive rewarding experiences may lead to addiction-like behaviors, and high engagement in virtual worlds may facilitate and perpetuate the avoidance of challenges in the offline environment. Further, time spent in virtual worlds may displace (reduce) other determinants of mental health, such as sleep rhythms and offline social capital. Importantly, individuals will differ in their uses of and psychological responses to the metaverse, resulting in heterogeneous impacts on their mental health. Their technological motivations, developmental stage, sociodemographic context, and prior mental health problems are some of the factors that may modify and frame the positive and negative effects of the metaverse on their mental health. In conclusion, as the metaverse is being scaffolded by the industry and by its users, there is a window of opportunity for researchers, clinicians, and people with lived experience to coproduce knowledge on its possible impacts on mental health and illness, with the hope of influencing policy-making, technological development, and counseling of patients.

## Introduction

In 2021, Facebook spent US $10 billion on its metaverse division, shipped 10 million virtual reality headsets, and rebranded itself as Meta [1]. The same year, 46 million users were logging onto the game platform Roblox every day, spending a total of 41 billion hours in this virtual world [2]. Concurrently, technology giants Apple, Microsoft, and Google have been developing their own metaverse-related infrastructures. While the idea of a metaverse is not new, the popularity of virtual worlds has boomed in the past few years, particularly during the COVID-19 pandemic [2,3].

The technology magazine *WIRED* defines the metaverse as an extension of “virtual reality—characterized by persistent virtual worlds that continue to exist even when you're not playing—as well as augmented reality that combines aspects of the digital and physical world” [4]. In virtual reality, users wear headsets to see a digital world in 3 dimensions. Through the avatars they embody, they can move in the virtual environment, have life-like interactions with other users, and control digital objects. In augmented reality, users see their real, physical environment, but digital elements are integrated through eyeglasses or a smartphone. Pokémon Go is an example of augmented reality: in this mobile game, the real-world locations of players are captured by their smartphone camera and overlaid with virtual creatures that they can interact with. Other devices combine elements of virtual and augmented reality, allowing users to simultaneously interact with virtual and real objects in “mixed reality” [5].

Although virtual worlds such as Pokémon Go and Roblox are already ubiquitous, the promise of the metaverse is to make these spaces more immersive and interconnected, and to extend their scope of application beyond play unto other aspects of daily life. Of late, there has been an emergence of virtual reality platforms such as VRChat, whose main purpose is to allow users to interact and socialize with others with their self-created 3D avatars. Increasingly, users of virtual worlds trade cryptocurrencies, which are digital currencies that function like money but are independent from states and central banks [6]. With cryptocurrencies, one can buy digital assets (eg, art, avatar apparel, and virtual estates) that are recorded on virtual ledgers as uniquely having one owner, and that are not interchangeable or duplicable; these assets are called nonfungible tokens [7]. Tying together a range of emerging technologies, metaverse platforms thus offer rich and vivid experiences, from games to new ways of socializing, working, learning, and shopping [4,8].

Will the metaverse impact mental health and illness? With every media revolution threatening to infiltrate people’s homes, whether the radio, TV, or the internet, there are concerns for mental health [9]. In line with popular views, a body of research points to potential harmful effects of digital media on depression, anxiety, and addiction-like behaviors [10-12]. However, in truth, cyberpsychology—the study of how humans and computers interact—paints a much more complex picture [13]. The mental health risks of digital media are far from universal and, at least in some cases, the adaptive use of technology may even contribute to improvement in short- and long-term well-being [14]. As interest in the possibilities of the metaverse continues to grow in the general population, we consider in the present viewpoint how this technology may impact mental health. We narratively present a selection of key articles from the literature on digital media to appraise the potential mental health risks and benefits of the metaverse and discuss directions for future research.

## Virtual Worlds and Mental Health

### Overview

Most research to date has examined exposure to digital media as a function of screen time, roughly defined as time spent on digital media and other screen devices. This body of literature generally shows that individuals who report greater screen time also report poorer mental health [15]. However, this association is likely to be confounded by other factors, such as preceding mental health conditions, occupational status, and lifestyle, to name a few. When individuals are studied as their own comparators, fluctuations in digital media use over time do not seem to predict changes in mental health [16-19], which goes against the hypothesis that higher screen time causes poorer mental health. However, the utility of screen time as an indicator of digital exposures is likely limited if the amount of time on digital media is not the primary mechanism by which digital media affect mental health. Media researchers have advocated for dropping the concept of screen time and focusing instead on more specific, theory-based measures of digital media exposures [14,16,20]. Limited research has investigated the mental health impacts of the metaverse, but many of the current platforms described as “metaverse” rely on virtual reality and are intended for internet-based gaming or socializing (see the *Introduction* section). The metaverse shares a number of characteristics with social media and video games: these include the possibility of interacting with other individuals in real time regardless of geographical location, the possibility of being anonymous, and the persistence of data over time [21,22]. Video games and virtual reality further afford vivid experiences that can be studied to better understand the potential psychological effects of immersion in the metaverse.

**Figure 1:**
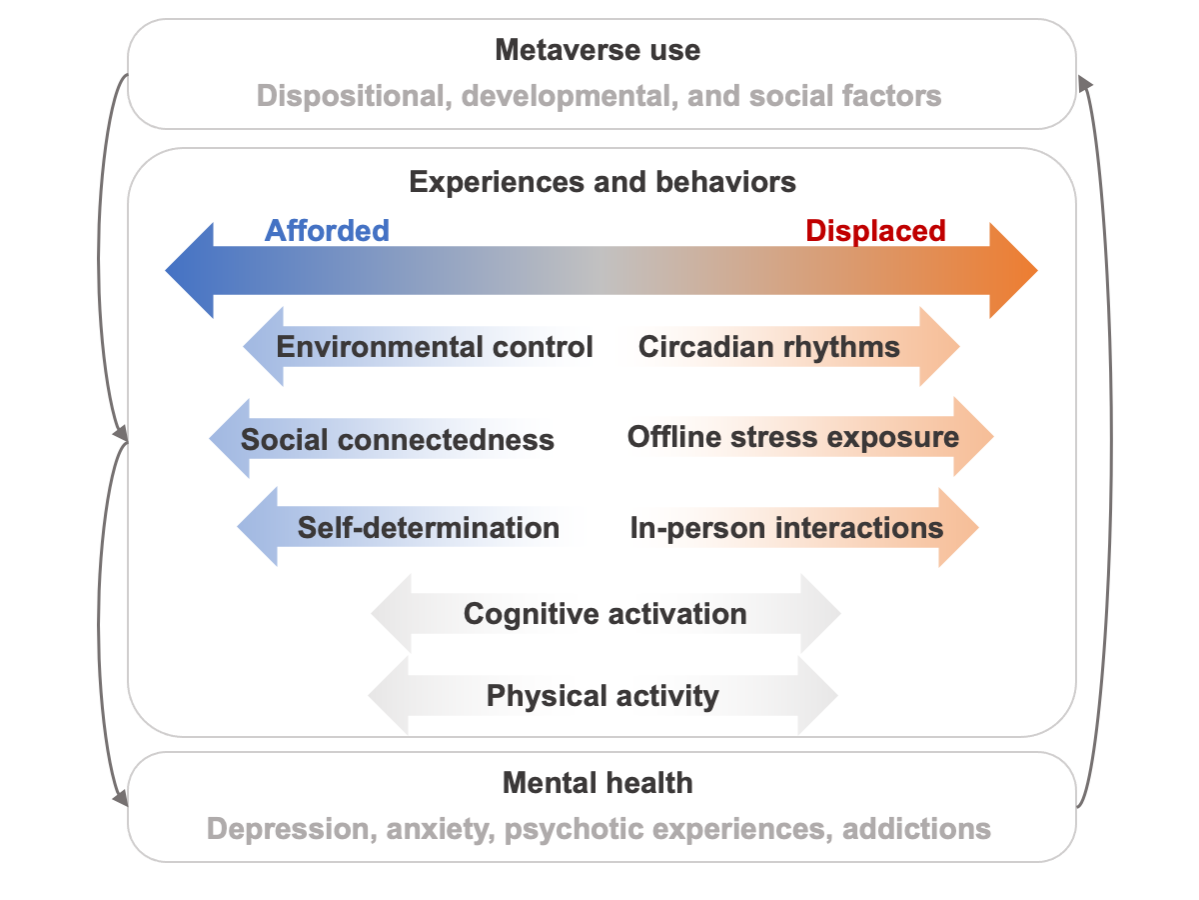
Interplay among metaverse use, experiences, behaviors, and mental health. Participation in the metaverse may afford certain experiences and behaviors relevant to mental health, while displacing others. The type of use, as well as individual and contextual factors, likely influence these effects. Together, these mechanisms subsequently influence mental health outcomes. In turn, mental states reciprocally influence interactions with the metaverse.

Thus, to anticipate how the metaverse will affect mental health, we can learn from the literature examining the mental health impacts of social media, video games, and virtual reality. From this state of knowledge, 2 broad categories of mechanisms, by which the metaverse may influence mental health, emerge: experiences or behaviors that are *afforded* by its use and experiences or behaviors that are *displaced* by it [9]. Below, we discuss these mechanisms in detail and summarize them in Figure 1.

### Affordances of the Metaverse

#### Environmental Control and Self-representation

Virtual worlds of the metaverse have the potential to afford users a number of experiences susceptible of impacting their mental health. This begins with the environment of the person, on which the metaverse affords a unique degree of control: users can easily choose and navigate the environment of their choice, or even self-design the space they are in. Virtual reality relaxation exploits this affordance by bringing the user into restorative, pleasant environments such as lakes, forests, or rivers. According to a recent systematic review, there is preliminary experimental evidence that virtual reality relaxation is effective for improving mood and anxiety, at least in the short term [23]. Conversely, exposure to social stressors in virtual environments is associated with higher levels of subjective distress and paranoia [24], illustrating the potential influence of virtual environmental exposures on mental health.

Virtual worlds also afford users to choose their avatars’ appearance and identity, which can influence how they perceive and interact with their surroundings. In an international survey of 142 regular users of video games, greater embodiment over an avatar was associated with lower awareness of bodily sensation during gaming sessions [25]. In a sample of 60 women from the UK general population, reducing avatar height during a virtual reality train ride was associated with increased levels of paranoia and negative social comparison [26]. Experimenting with self-representation in the metaverse may also participate in youth development: some authors have suggested that during adolescence, conducting identity experiments on the internet could help in the development of social competence [27]. However, strong investment in self-representation in virtual spaces could also cause harms, such as dissatisfaction with one’s physical body. This risk has been documented in studies of social media usage, showing that people’s investment in receiving feedback on their self-portrait photographs, and their comparisons with the photographs of others, may contribute to body dissatisfaction and drive for thinness [28,29].

#### Cognitive Activation and Physical Activity

The design of metaverse contents may be harnessed toward activating a person’s cognitive functions in beneficial ways. Many games have been developed to encourage players to use their cognitive skills to solve problems [30]. In a randomized controlled trial of 72 US adults with major depressive disorder, a video game–based intervention was effective in improving sustained attention and global cognitive functioning compared with an active control [31]. The embodied navigation of virtual worlds, enabled by sensors that capture the user’s movement in physical space, can also promote physical activity. This is the case of video games involving physical activity (“exergames”), which can produce significant improvements in cognitive functioning among clinical and nonclinical populations, according to a meta-analysis of randomized controlled trials [32].

#### Social Connectedness

Interpersonal relationships could be developed and maintained through the metaverse. The capacity to connect with others regardless of physical location enhance individuals’ opportunities to join communities with shared interests, values, or identities. Members of marginalized groups and people living with mental health problems may benefit from these platforms for sharing knowledge and obtaining peer support [33-35]. Importantly, internet-based interactions can be experienced as meaningful. In an international survey of people using massively multiplayer online role-playing games (MMORPGs), respondents frequently reported high-quality relationships in their games, which sometimes transformed into friendships or romantic partnerships in the physical world [36,37]. MMORPG users also commonly play with their offline friends or romantic partners [37,38], suggesting that virtual worlds can foster both the formation of new relationships and the enhancement of existing ones. More research is needed to understand the extent to which these social affordances impact mental health, but arguably, they may be beneficial if they decrease a person’s loneliness and increase their social capital [39].

#### Self-determination

Experiences of autonomy and competence in virtual worlds can fulfill the need for self-determination. In a survey of 672 MMORPG players, positive experiences associated with intensive gaming involvement included feelings of achievement, positive anticipation and stress (eg, “adrenaline rush”), enjoyable repetition and routine, positive social obligation, satisfying labor, increased confidence, and positive distraction, to name a few [40]. In samples of college students playing video games in experimental settings, experiences of autonomy and competence while playing were associated with gaming motivation and enjoyment [41]. For people experiencing disability, functional impairments, or social adversity in the context of mental health problems, digital spaces that operate under different parameters than the offline, physical world may thus provide invaluable opportunities for fulfilling the psychological needs related to self-determination.

#### Addictions

Because they repetitively afford rewarding experiences, virtual worlds can also be the object of addiction-like behaviors. Gaming disorder, which made its entry in the 11th revision of the International Classification of Diseases, captures how this may translate into mental health problems [42]. Gaming disorder is defined by persistent or recurrent gaming behavior, manifesting as impaired control over gaming, increased priority given to gaming (at the expense of other interests and activities), and continuation or escalation of gaming despite the occurrence of negative consequences. A meta-analysis situates the global prevalence of gaming disorder at 2%-3%, with the most common type being internet-based rather than offline gaming [43]. Attributing problematic gaming behaviors as constitutive of a mental disorder is subject to controversy [10,44]. But at the minimum, epidemiological and cross-cultural data show that some players engage in gaming in ways that negatively impact their well-being and functioning [43,45]. In the metaverse, immersive technologies broaden the scope of daily-life activities that can be performed digitally, raising the possibility of even greater absorption into virtual worlds and greater interference with functioning. Addiction is a particular concern considering that companies have a financial incentive to maximize virtual worlds’ capacity to captivate users—something that can be facilitated by passively monitoring users’ behaviors in the metaverse and by customizing their environment accordingly [46].

### Displacement of Offline Activities

#### Circadian Rhythms

High engagement in the metaverse could be at the expense of other activities in the offline environment. According to the displacement hypothesis [47], excessive use of digital media causes harm to the individual by displacing time spent on other activities that are beneficial for mental health. Notably, participating in virtual worlds in the evening or at night may impair sleep rhythms because of delayed or reduced sleep time. According to a recent narrative review, previous observational studies support an association between greater use of screen-based media and poorer sleep outcomes in adolescents; however, experimental research does not consistently show that screen use has causal effects on sleep [48]. Three mechanisms could be responsible for the putative effects of screen use on sleep: disruption of the neurobiology of sleep due to light emitted by the screen devices, delay of sleep onset or reduction of total sleep time because of time spent on the screen device, and psychological stimulation caused by digital media content [48]. More evidence is needed to determine the causal effects of screens on sleep and whether the degree of immersion afforded by the metaverse will amplify these effects.

#### Offline Stress Exposure

Individuals may participate in virtual worlds to avoid or escape from anxiety-provoking situations of the offline world. In web-based surveys, avoidance and escapism are relatively commonly reported by video gamers as part of their motivations for gaming [36,45,49]. For example, in an international survey of internet-based gamers, participants endorsed some degree of “gaming to avoid challenges in [their] life rather than deal with them directly” (average item score of 2.46 on a scale of 1 to 5)—a finding that was consistent across North America, Europe, and China [45]. Recurrent avoidance of stressful situations may prevent a person from seeking to solve the problems or from learning to tolerate the stressors they face. However, it has been suggested that escapism through video gaming can also be an adaptive coping strategy that helps regulate or restore mood following exposure to stressful situations [50,51]. A systematic review of surveys conducted among video gamers found that escapism is cross-sectionally associated with both negative outcomes (eg, problematic video gaming, social anxiety, and loneliness) and positive outcomes (eg, social connection, enjoyment, and psychological well-being) [49]. Longitudinal studies are needed to better understand how escapism in the metaverse interacts with and affects a person’s mental health problems over time.

#### In-Person Interactions

A related topic is how the potential displacement of in-person social interactions toward their digital equivalent influences mental health. As highlighted above, there is evidence that internet-based gaming can foster meaningful social interactions both digitally and offline. However, if high social involvement in the metaverse is accompanied by limited social interaction in the offline world, the net result may be an increase in social anxiety and a decrease in social skills in offline contexts [39]. This could explain why internet addiction was associated with a prospective increase in loneliness in a convenience sample of 361 college students in Hong Kong [52]. Potentially, for individuals living with social anxiety or low social skills, achieving connectedness through virtual worlds can simultaneously be a compensatory strategy and a perpetuating factor that constrains exposure to in-person social interactions.

### The Importance of Individual and Contextual Factors

#### Overview

The effects of virtual worlds on mental health likely depend on individual and contextual factors. To begin with, virtual worlds and the technologies that support them differ in their features and affordances, including those discussed above: control of the avatar and environment, cognitive stimulation, social connectedness, and the satisfaction of other psychological needs. Users also have freedom in how they take advantage of virtual worlds’ possibilities. Differences in digital media uses, and their relevance for mental health correlates, are frequently observed in the scientific literature. For example, in an experience sampling study of 44 adults with and those without psychosis in the United Kingdom, posting about feelings and venting on social media predicted higher subsequent paranoia, while posting about daily activities predicted lower paranoia [53]. Although such findings are correlational and subject to confounding, they reflect the heterogeneity of uses and mental health correlates for a given technology. This heterogeneity is perhaps best understood in terms of individual or contextual factors. Borrowing on the terminology of Valkenburg and Peter [54], factors that modify and frame the mental health impacts of digital media can be organized in 3 categories: dispositional, developmental, and social.

#### Dispositional and Developmental Factors

At the dispositional level, individuals’ motivations and interests will likely influence how they engage with the metaverse. Among US school-aged children, boys generally report greater use of video games than girls, whereas girls report greater use of social media [55]. In an international longitudinal study of video game players, a person’s increase in intrinsic motivation for gaming was associated with a subsequent improvement in their affect and life satisfaction; conversely, an increase in extrinsic motivation (ie, feeling pressured to play) was association with a deterioration of the same outcomes [16]. At the developmental level, age may moderate the mental health outcomes of metaverse usage as a function of the person’s developmental needs and sensitivities, as well as age-dependent differences in motivations and profiles of use. To illustrate, early childhood is a crucial period for cognitive development, and there is tentative evidence from a Canadian study that a child’s increase in screen time is associated with a decrease in their performance on developmental screening tests between ages of 24 and 60 months [56]. Adolescence is marked by social and identity development, and teens may be particularly sensitive to social acceptance and rejection in virtual worlds [57]. Some authors have also argued that digital experiences have the potential to contribute to agency, connectedness, and storytelling capacities that foster youth’s identity development [58].

#### Social Factors

Social and economic factors are sources of inequalities in the access to metaverse-related technologies. Lower income or economic development is associated with reduced access to internet-connected devices, and in many emerging countries, women have lower access to the internet than men [59]. Given the cost of virtual reality headsets and related technologies, the rise in popularity of metaverse platforms may contribute to widening digital inequalities. Other sources of digital inequalities include older age, as older adults tend to be less autonomous and experienced in the use of new technologies [59], and physical disabilities, which to date have received little attention in the development of virtual reality hardware [60]. Digital inequalities are important to consider in the design of policies and the organization of services relevant to mental health care: these inequalities may translate into unequal access to digital mental health interventions, as well to health information and economic opportunities—all of which are determinants of mental health and recovery.

Virtual worlds are also a space where people can be exposed to bullying and discrimination. Exposure to cyberbullying is relatively common among the youth and has been associated with higher subsequent risk of psychological distress, suicidal ideations, and delinquency [61]. People in virtual worlds may feel emboldened to enact discriminatory and aggressive behaviors, as suggested by reports of sexual harassment, racism, homophobia, and transphobia within some video game communities [59,62,63]. Considering that 3D embodiment over an avatar, and its interactions with a virtual environment, can make internet-based interactions particularly vivid, one question that appears relevant for future research is to consider whether the metaverse risks magnifying the psychological impacts of internet-based bullying and discrimination.

People’s offline environment may further influence how they engage with virtual worlds and their propensity for using them in a problematic, addiction-like manner. Socioeconomic disparities and other forms of social adversity could increase people’s propensity for problematic metaverse use, for example, by motivating them to escape their stressful environment, or by constraining their access to alternative activities in their neighborhoods. Nagata et al [64] proposed that these effects of social adversity may explain, to some extent, why higher levels of problematic video game use are reported by adolescents in the United States who identify as Native American, Black, or Latinx, and by those with lower parental educational attainment. Conversely, positive social interactions in the offline environment could be protective against the risk of problematic metaverse use. Illustrating this, a study of 250 MMORPG players found that higher levels of cultural consonance (ie, feeling successful in conventional society) and playing with offline friends were both cross-sectionally associated with lower levels of problematic gaming [38,65]. Similarly, in 2 samples of adult video game players, higher satisfaction of psychological needs in the offline world was cross-sectionally associated with lower levels of internet gaming disorder [66]. Young people’s interactions with their parents may be particularly important in shaping how they engage with metaverse technologies. For example, a community-based study of 2974 children and adolescents in Singapore showed that higher parent-child closeness was subsequently associated with a decrease in their levels of pathological video gaming after 1 year [67]. Interactions with friends, family, and society in the offline world are thus relevant to understand individuals’ engagement in virtual worlds. Together, these findings illustrate how individual and contextual factors, spanning dispositional, developmental, and social dimensions, may influence the mental health impacts of engagement in the metaverse.

## Conclusions: Immersion in the Metaverse

When considered critically, the last 2 decades of research do not demonstrate universal harms of digital media on the mental health of the general population. The state of knowledge reveals many ways in which digital media may benefit or harm a person’s well-being, whether as a function of technological, individual, or contextual factors. But popular concerns persist and will almost certainly grow if the metaverse fulfills its commercial and social ambitions. Perhaps what sets the metaverse apart from previous technologies is the greater immersivity it affords, compared with traditional social media and video game devices. Immersion seems to be a factor that could amplify many of the mental health impacts described above: with greater immersion may come greater displacement of bodily awareness, greater embodiment over one’s avatar, greater sense of copresence with internet-based friends, and generally more vivid experiences. In turn, these features and experiences are likely to shape the motivations, affordances, and mental health risks associated with metaverse use.

It is too early to indicate whether the metaverse will broadly be a greater risk or benefit to mental health than previous digital media. Undoubtedly, virtual worlds are unprecedented in the scope of experiences and behaviors they can afford, and as such, in their potential to take a greater place in our daily lives. To adequately counsel patients and guide policies around the development and implementation of the metaverse, there is a need for timely and nuanced research on its opportunities and risks for mental health [68,69]. Arguably, previous research on digital media has often failed to achieve timely and nuanced knowledge production, partly due to the lack of clear theoretical frameworks and robust empirical methods [9,20]. Following these lessons, we believe a starting point is to examine the experiences and behaviors that are afforded and displaced by the metaverse, as well as the interplay of these effects with dispositional, developmental, and social factors. Collaboration among clinicians, researchers, individuals with lived experience, technology users, the industry, and other stakeholders will be crucial to successfully generate and translate this new body of knowledge. While metaverse technologies are in the process of being scaffolded and disseminated, psychiatry has a window of opportunity to think through conceptually and to strategically examine their risks and benefits for mental health.

## Abbreviations

MMORPG: massively multiplayer online role-playing game

## References

[1] Isaac M (2022). Meta spent $10 billion on the metaverse in 2021, dragging down profit. The New York Times.

[2] Stanton R (2022). If Roblox's daily users were a country, it would be bigger than Canada. PC Gamer.

[3] Madigan S, Eirich R, Pador P, McArthur BA, Neville RD (2022). Assessment of changes in child and adolescent screen time during the COVID-19 pandemic: a systematic review and meta-analysis. JAMA Pediatr.

[4] Ravenscraft E (2022). What Is the Metaverse, Exactly?. Wired.

[5] Speicher M, Hall B, Nebeling M (2019). What is Mixed Reality?.

[6] Maese V, Avery A, Naftalis B, Wink S, Valdez Y (2016). Cryptocurrency: A Primer. Banking Law Journal.

[7] Chohan U (2021). Non-Fungible Tokens: Blockchains, Scarcity, and Value. SSRN J.

[8] Kye B, Han N, Kim E, Park Y, Jo S (2021). Educational applications of metaverse: possibilities and limitations. J Educ Eval Health Prof.

[9] Orben A (2020). The Sisyphean cycle of technology panics. Perspect Psychol Sci.

[10] Griffiths MD, Kuss DJ, Lopez-Fernandez O, Pontes HM (2017). Problematic gaming exists and is an example of disordered gaming. J Behav Addict.

[11] Twenge JM, Joiner TE, Rogers ML, Martin GN (2017). Increases in depressive symptoms, suicide-related outcomes, and suicide rates among U.S. adolescents after 2010 and links to increased new media screen time. Clin Psychol Sci.

[12] Boers E, Afzali MH, Conrod P (2020). Temporal associations of screen time and anxiety symptoms among adolescents. Can J Psychiatry.

[13] Attrill-Smith A, Fullwood C, Keep M, Kuss D (2019). The Oxford Handbook of Cyberpsychology.

[14] Vanden AM (2021). Digital wellbeing as a dynamic construct. Commun Theory.

[15] Wang X, Li Y, Fan H (2019). The associations between screen time-based sedentary behavior and depression: a systematic review and meta-analysis. BMC Public Health.

[16] Vuorre M, Johannes N, Magnusson K, Przybylski AK (2022). Time spent playing video games is unlikely to impact well-being. R Soc Open Sci.

[17] Houghton S, Lawrence D, Hunter SC, Rosenberg M, Zadow C, Wood L, Shilton T (2018). Reciprocal relationships between trajectories of depressive symptoms and screen media use during adolescence. J Youth Adolesc.

[18] Coyne SM, Rogers AA, Zurcher JD, Stockdale L, Booth M (2020). Does time spent using social media impact mental health? An eight year longitudinal study. Comput Hum Behav.

[19] Paquin V, Philippe F, Shannon H, Guimond S, Ouellet-Morin I, Geoffroy MC Associations between digital media use and psychotic experiences in young adults of Quebec, Canada: a longitudinal study. PsyArXiv..

[20] Kaye LK, Orben A, Ellis DA, Hunter SC, Houghton S (2020). The conceptual and methodological mayhem of "Screen Time". Int J Environ Res Public Health.

[21] McCain J, Morrison K, Ahn S, Attrill-Smith A, Fullwood C, Keep M, Kuss DJ (2018). Video Games and Behavior Change. The Oxford Handbook of Cyberpsychology.

[22] Evans SK, Pearce KE, Vitak J, Treem JW (2016). Explicating affordances: a conceptual framework for understanding affordances in communication research. J Comput-Mediat Comm.

[23] Riches S, Azevedo L, Bird L, Pisani S, Valmaggia L (2021). Virtual reality relaxation for the general population: a systematic review. Soc Psychiatry Psychiatr Epidemiol.

[24] Veling W, Pot-Kolder R, Counotte J, van Os Jim, van der Gaag Mark (2016). Environmental social stress, paranoia and psychosis liability: a virtual reality study. Schizophr Bull.

[25] Swinkels LM, Veling H, van Schie HT (2021). Playing videogames is associated with reduced awareness of bodily sensations. Comput Hum Behav.

[26] Freeman D, Evans N, Lister R, Antley A, Dunn G, Slater M (2014). Height, social comparison, and paranoia: an immersive virtual reality experimental study. Psychiatry Res.

[27] Valkenburg P, Peter J (2008). Adolescents' identity experiments on the internet. Commun Res.

[28] Au ES, Cosh SM (2022). Social media and eating disorder recovery: an exploration of Instagram recovery community users and their reasons for engagement. Eat Behav.

[29] Butkowski CP, Dixon TL, Weeks K (2019). Body surveillance on Instagram: examining the role of selfie feedback investment in young adult women’s body image concerns. Sex Roles.

[30] Pallavicini F, Ferrari A, Mantovani F (2018). Video games for well-being: a systematic review on the application of computer games for cognitive and emotional training in the adult population. Front Psychol.

[31] Keefe RS, Cañadas Elena, Farlow D, Etkin A (2022). Digital intervention for cognitive deficits in major depression: a randomized controlled trial to assess efficacy and safety in adults. Am J Psychiatry.

[32] Stanmore E, Stubbs B, Vancampfort D, de Bruin ED, Firth J (2017). The effect of active video games on cognitive functioning in clinical and non-clinical populations: a meta-analysis of randomized controlled trials. Neurosci Biobehav Rev.

[33] Ali K, Farrer L, Gulliver A, Griffiths KM (2015). Online peer-to-peer support for young people with mental health problems: a systematic review. JMIR Ment Health.

[34] Gauld C, Maquet J, Micoulaud-Franchi J, Dumas G (2022). Popular and scientific discourse on autism: representational cross-cultural analysis of epistemic communities to inform policy and practice. J Med Internet Res.

[35] Saha K, Kim SC, Reddy MD, Carter AJ, Sharma E, Haimson OL, DE Choudhury Munmun (2019). The Language of LGBTQ+ Minority Stress Experiences on Social Media. Proc ACM Hum Comput Interact.

[36] Yee N (2006). The demographics, motivations, and derived experiences of users of massively multi-user online graphical environments. Presence: Teleoperators Virtual Environ.

[37] Yee N, Schroeder R, Axelsson AS (2006). The psychology of massively multi-user online role-playing games: motivations, emotional investment, relationships and problematic usage. Avatars at Work and Play: Collaboration and Interaction in Shared Virtual Environments.

[38] Snodgrass JG, Lacy MG, Francois Dengah H, Fagan J (2011). Enhancing one life rather than living two: playing MMOs with offline friends. Comput Hum Behav.

[39] Nowland R, Necka EA, Cacioppo JT (2018). Loneliness and social internet use: pathways to reconnection in a digital world?. Perspect Psychol Sci.

[40] Snodgrass JG, Dengah HF, Lacy MG, Bagwell A, Van Oostenburg M, Lende D (2017). Online gaming involvement and its positive and negative consequences: a cognitive anthropological “cultural consensus” approach to psychiatric measurement and assessment. Comput Hum Behav.

[41] Ryan RM, Rigby CS, Przybylski A (2006). The motivational pull of video games: a self-determination theory approach. Motiv Emot.

[42] ICD-11 for Mortality and Morbidity Statistics (ICD-11 MMS). ICD-11 for Mortality and Morbidity Statistics (Version : 02/2022).

[43] Stevens MW, Dorstyn D, Delfabbro PH, King DL (2021). Global prevalence of gaming disorder: a systematic review and meta-analysis. Aust N Z J Psychiatry.

[44] Aarseth E, Bean AM, Boonen H, Colder Carras Michelle, Coulson M, Das D, Deleuze J, Dunkels E, Edman J, Ferguson CJ, Haagsma MC, Helmersson Bergmark Karin, Hussain Z, Jansz J, Kardefelt-Winther D, Kutner L, Markey P, Nielsen RKL, Prause N, Przybylski A, Quandt T, Schimmenti A, Starcevic V, Stutman G, Van Looy Jan, Van Rooij Antonius J (2017). Scholars' open debate paper on the World Health Organization ICD-11 Gaming Disorder proposal. J Behav Addict.

[45] Snodgrass JG, Zhao W, Lacy MG, Zhang S, Tate R (2019). Distinguishing core from peripheral psychiatric symptoms: addictive and problematic internet gaming in North America, Europe, and China. Cult Med Psychiatry.

[46] Bailenson J (2018). Protecting nonverbal data tracked in virtual reality. JAMA Pediatr.

[47] Neuman SB (1988). The displacement effect: assessing the relation between television viewing and reading performance. Read Res Q.

[48] Hale L, Li X, Hartstein LE, LeBourgeois MK (2019). Media use and sleep in teenagers: what do we know?. Curr Sleep Medicine Rep.

[49] Hussain U, Jabarkhail S, Cunningham GB, Madsen JA (2021). The dual nature of escapism in video gaming: a meta-analytic approach. Comput Hum Behav Rep.

[50] Giardina A, Starcevic V, King DL, Schimmenti A, Di Blasi M, Billieux J (2021). Research directions in the study of gaming-related escapism: a commentary to Melodia, Canale, and Griffiths (2020). Int J Ment Health Addiction.

[51] Kardefelt-Winther D (2017). Conceptualizing internet use disorders: addiction or coping process?. Psychiatry Clin Neurosci.

[52] Yao MZ, Zhong Z (2014). Loneliness, social contacts and internet addiction: a cross-lagged panel study. Comput Hum Behav.

[53] Berry N, Emsley R, Lobban F, Bucci S (2018). Social media and its relationship with mood, self-esteem and paranoia in psychosis. Acta Psychiatr Scand.

[54] Valkenburg PM, Peter J (2013). The differential susceptibility to media effects model. J Commun.

[55] Bagot KS, Tomko RL, Marshall AT, Hermann J, Cummins K, Ksinan A, Kakalis M, Breslin F, Lisdahl KM, Mason M, Redhead JN, Squeglia LM, Thompson WK, Wade T, Tapert SF, Fuemmeler BF, Baker FC (2022). Youth screen use in the ABCD® study. Dev Cogn Neurosci.

[56] Madigan S, Browne D, Racine N, Mori C, Tough S (2019). Association between screen time and children's performance on a developmental screening test. JAMA Pediatr.

[57] Crone EA, Konijn EA (2018). Media use and brain development during adolescence. Nat Commun.

[58] Granic I, Morita H, Scholten H (2020). Beyond screen time: identity development in the digital age. Psychological Inquiry.

[59] Robinson L, Schulz J, Blank G, Ragnedda M, Ono H, Hogan B, Mesch G, Cotten S, Kretchmer S, Hale T, Drabowicz T, Yan P, Wellman B, Harper M, Quan-Haase A, Dunn H, Casilli A, Tubaro P, Carvath R, Chen W, Wiest J, Dodel M, Stern M, Ball C, Huang K, Khilnani A (2020). Digital inequalities 2.0: Legacy inequalities in the information age. First Monday.

[60] Egliston B, Carter M (2021). Critical questions for Facebook’s virtual reality: data, power and the metaverse. Internet Policy Rev.

[61] Kim S, Kimber M, Boyle MH, Georgiades K (2019). Sex differences in the association between cyberbullying victimization and mental health, substance use, and suicidal ideation in adolescents. Can J Psychiatry.

[62] Kowert R, Martel A, Swann B (2022). Not just a game: Identity fusion and extremism in gaming cultures. Front Commun.

[63] Lopez-Fernandez O, Williams A, Griffiths M, Kuss D (2019). Female gaming, gaming addiction, and the role of women within gaming culture: a narrative literature review. Front Psychiatry.

[64] Nagata JM, Singh G, Sajjad OM, Ganson KT, Testa A, Jackson DB, Assari S, Murray SB, Bibbins-Domingo K, Baker FC (2022). Social epidemiology of early adolescent problematic screen use in the United States. Pediatr Res.

[65] Snodgrass J, Lacy M, Dengah HJF, Fagan J (2011). Cultural consonance and mental wellness in the World of Warcraft: online games as cognitive technologies of ‘Absorption-Immersion’. Cogn Technol.

[66] Bender PK, Gentile DA (2020). Internet gaming disorder: Relations between needs satisfaction in-game and in life in general. Psychol Pop Media.

[67] Choo H, Sim T, Liau AKF, Gentile DA, Khoo A (2014). Parental influences on pathological symptoms of video-gaming among children and adolescents: a prospective study. J Child Fam Stud.

[68] Benrimoh D, Chheda FD, Margolese HC (2022). The best predictor of the future-the metaverse, mental health, and lessons learned from current technologies. JMIR Ment Health.

[69] Usmani SS, Sharath M, Mehendale M (2022). Future of mental health in the metaverse. Gen Psychiatr.

